# Supplementary figures and images for: Olfactory receptor accessory proteins play crucial roles in receptor function and gene choice
Source: eLife. 2017 Mar 6;6:e21895. doi: 10.7554/eLife.21895 (PMC5362263; doi:10.7554/eLife.21895)

Figure 2-figure supplement 1

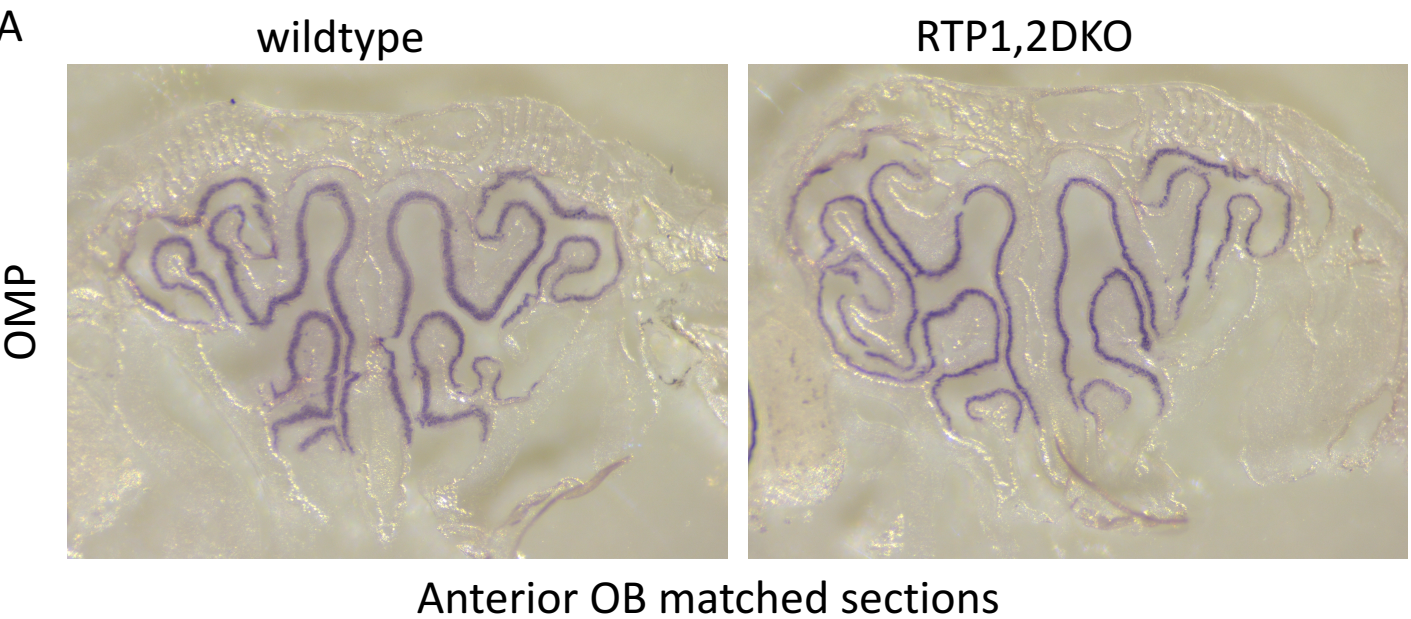

Supplement: Supplementary file 1. — The number of sequence reads that map each annotated gene in RNA-Seq from three wild-type and 3 RTP1,2DKO mice. FDR is calculated against the entire data set and the fold change (logFC) is displayed as the log (average wild-type reads/ average RTP1,2DKO reads). DOI: http://dx.doi.org/10.7554/eLife.21895.024 [file elife-21895-supp1.pdf]

Figure 9-figure supplement 1

A

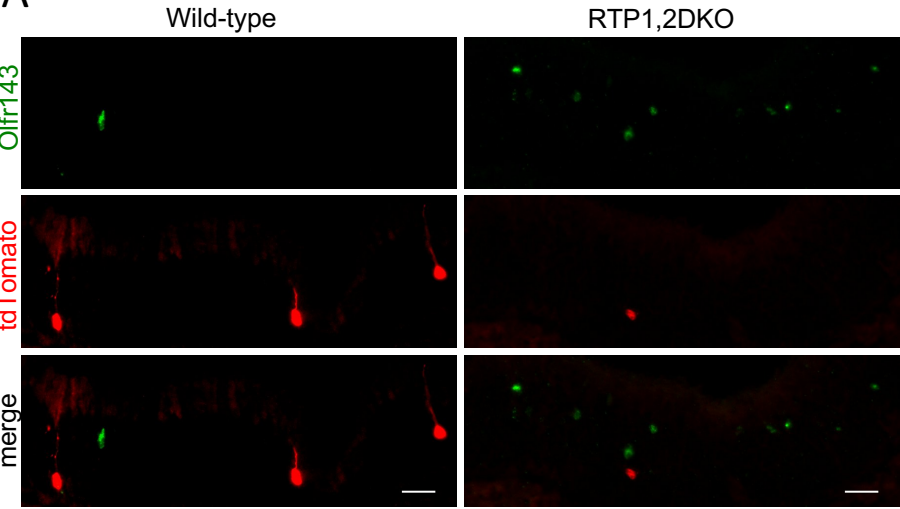

B

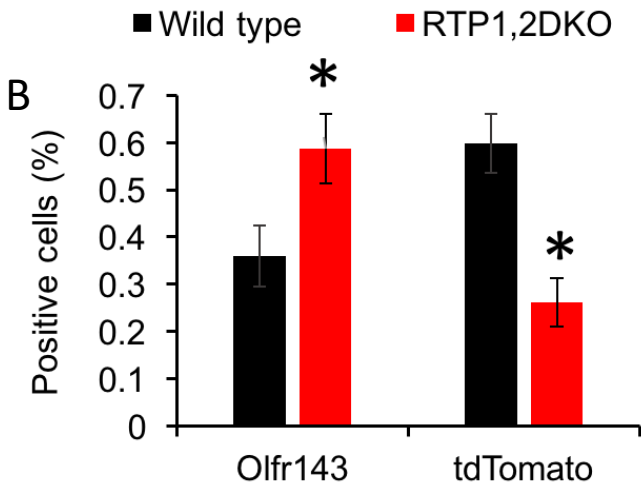

C

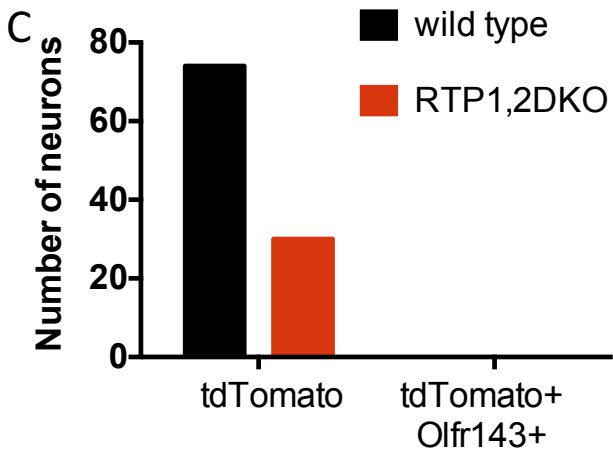

Supplement: Supplementary file 3. — Results of one-way ANOVA and Tukey’s post hoc tests DOI: http://dx.doi.org/10.7554/eLife.21895.026 [file elife-21895-supp3.pdf]
